# Transcriptomic Analysis Revealed Key Defense Genes and Signaling Pathways Mediated by the *Arabidopsis thaliana* Gene *SAD2* in Response to Infection with *Pseudomonas syringae* pv. Tomato DC3000

**DOI:** 10.3390/ijms24044229

**Published:** 2023-02-20

**Authors:** Sha Li, Tiantian Shi, Mingjie Lyu, Rui Wang, Andi Xu, Luoying Chen, Rong Luo, Yinglu Sun, Xiaoying Guo, Jun Liu, Huan Wang, Ying Gao

**Affiliations:** 1 Biotechnology Research Institute, Chinese Academy of Agricultural Sciences (CAAS), Beijing 100081, China; 2 National Key Facility for Crop Gene Resources and Genetic Improvement (NFCRI), Institute of Crop Sciences, Chinese Academy of Agriculture Sciences (CAAS), Beijing 100081, China; 3 Institute of Germplasm Resources and Biotechnology, Tianjin Academy of Agricultural Sciences, Tianjin 300112, China; 4 College of Horticulture and Landscape Architecture, Tianjin Agricultural University, Tianjin 300392, China; 5 Chengdu National Agricultural Science and Technology Center, Chengdu 610213, China

**Keywords:** Arabidopsis, SAD2, *Pst* DC3000, pathogen defense, RNA-Seq, DEG

## Abstract

Nucleocytoplasmic transport receptors play key roles in the nuclear translocation of disease resistance proteins, but the associated mechanisms remain unclear. The *Arabidopsis thaliana* gene *SAD2* encodes an importin β-like protein. A transgenic Arabidopsis line overexpressing *SAD2* (OESAD2/Col-0) showed obvious resistance to *Pseudomonas syringae* pv. tomato DC3000 (*Pst* DC3000) compared to the wild type (Col-0), but the knockout mutant *sad2-5* was susceptible. Transcriptomic analysis was then performed on Col-0, OESAD2/Col-0, and *sad2-5* leaves at 0, 1, 2, and 3 days post-inoculation with *Pst* DC3000. A total of 1825 differentially expressed genes (DEGs) were identified as putative biotic stress defense genes regulated by SAD2, 45 of which overlapped between the *SAD2* knockout and overexpression datasets. Gene Ontology (GO) analysis indicated that the DEGs were broadly involved in single-organism cellular metabolic processes and in response to stimulatory stress. Kyoto Encyclopedia of Genes and Genomes (KEGG) biochemical pathway analysis revealed that many of the DEGs were associated with the biosynthesis of flavonoids and other specialized metabolites. Transcription factor analysis showed that a large number of ERF/AP2, MYB, and bHLH transcription factors were involved in SAD2-mediated plant disease resistance. These results provide a basis for future exploration of the molecular mechanisms associated with SAD2-mediated disease resistance and establish a set of key candidate disease resistance genes.

## 1. Introduction

Plant pathogens are a serious threat to global food production, reducing crop yield and quality [1]. With increases in the human population and decreases in available cultivated land, methods of improving grain yield and quality have become a key focus of scientific research [2,3]. Previous studies have revealed plant disease resistance (R) genes and elucidated molecular mechanisms associated with plant disease resistance [4,5]. This research not only provides a basis for fundamental understanding of plant pathogen defense mechanisms but also lays a theoretical foundation for the more efficient improvement of crop yield and quality traits.

Because they are immobile, plants cannot avoid unfavorable environments or hazards during growth and development; they have therefore developed complex defense mechanisms [6,7]. To survive, plants must effectively sense and defend against invading pathogens [8]. To this end, they have evolved two primary layers of defense. The first layer is pathogen-associated molecular pattern (PAMP)-triggered immunity (PTI) [6,9]. PTI is triggered when PAMPs or microbe-associated molecular patterns (MAMPs) are sensed by pattern recognition receptors (PRRs) on the outer surface of plant cells. PRRs bind to co-receptors to activate a series of downstream immune responses, which initiate defense responses. Such responses include reactive oxygen species (ROS) bursts, mitogen-activated protein kinase (MAPK) activation, and defense-related gene expression. PTI can effectively prevent the invasion of most pathogenic bacteria, but the associated defense responses are typically short-lived [10,11]. To circumvent plant immune responses, pathogens produce effector proteins that are delivered directly into plant cells, bypassing PTI and making plants susceptible to disease [12,13]. In response, plants have acquired additional intracellular receptors and sensors called R proteins. These proteins recognize pathogen effectors to initiate a second layer of defense called effector-triggered immunity (ETI). ETI responses are generally stronger and more persistent than PTI responses and often lead to localized cell death [14,15]. Recognition of pathogen effectors by R proteins triggers nuclear translocation of disease-resistance proteins, thereby activating defense signals [16,17]. Nucleocytoplasmic transport receptors play key roles in this process.

In plants, a variety of nucleocytoplasmic transport pathways, including nuclear export of mRNA and nuclear import of immune-related proteins, are involved in innate immunity. Many macromolecules involved in defense responses, such as RNA and proteins, must be transferred between the cytoplasm and the nucleus to properly perform their functions. Nucleocytoplasmic transport mediates key processes: immune receptor activation; defense signal generation; intracellular defense protein transport to pathogen inoculation sites; and stimulation of programmed cell death [18,19,20]. An increasing number of studies therefore indicate that nucleocytoplasmic transport receptors play key roles in pathogen resistance [21,22,23,24,25]. Importin β is a nuclear import receptor that was first discovered in human cells; it forms a complex with importin α, which carries a substrate, to import the substrate into the nucleus [10]. Subsequently, multiple nuclear transport receptors that are homologous to importin β have been identified and classified as importin β-like proteins. Many functional disease-resistance proteins are transported to the nucleus with the assistance of importin β family proteins to induce defense responses, meaning that importin β family proteins play important roles in defense against disease [26,27,28]. Mutating the Arabidopsis gene encoding the importin β superfamily member *MOS14* causes the splicing of two R genes, *SNC1* and *RPS4*, thus impairing SNC1- and RPS4-mediated disease resistance. These findings indicate that the nuclear import of MOS14 is required for the correct splicing of *SNC1* and *RPS4* [26]. In *Arabidopsis thaliana*, SAD2 is encoded by the importinβ-like gene *AT2G31660* and contains a canonical importin β domain [29,30,31]. In a previous study, we screened an Arabidopsis mutant, *sad2-5*, which harbors a transfer (T)-DNA insertion in the 18th exon of *SAD2*. In response to fumonisin-B1 (FB1) treatment, *sad2-5* showed a clear cell death phenotype and abnormal H_2_O_2_ accumulation. These results suggested that the nuclear transport protein SAD2 plays an important role in calcium and H_2_O_2_-mediated programmed cell death [32].

In the present study, we found that a transgenic Arabidopsis line overexpressing *SAD2* (OESAD2/Col-0) was resistant to *Pseudomonas syringae* pv. tomato (*Pst*) DC3000 infection, whereas the *SAD2* knockout mutant (*sad2-5*) was susceptible. This indicated that *SAD2* may mediate the bacterial pathogen defense response in Arabidopsis. To identify candidate genes that are regulated by *SAD2* and involved in response to *Pst* DC3000, we analyzed the transcriptomes of wild-type (Col-0), *sad2-5*, and OESAD2/Col-0 plants at multiple time points after treatment with *Pst* DC3000 to establish differentially expressed genes (DEGs) between the genotypes. Through analysis of enriched Gene Ontology (GO) functional annotations and Kyoto Encyclopedia of Genes and Genomes (KEGG) metabolic pathways among the DEGs, we identified key stress-response genes regulated by *SAD2*, metabolic pathways associated with disease resistance, and disease-resistance signal transduction pathways. This study provides a theoretical reference for further exploration of resistance genes involved in the nuclear cytoplasmic transport pathway and lays the foundation for genetic improvement of crop plants through the introgression of the candidate genes identified here.

## 2. Results

### 2.1. SAD2 Expression Increased Resistance to Pst DC3000 Infection

**Figure 1:**
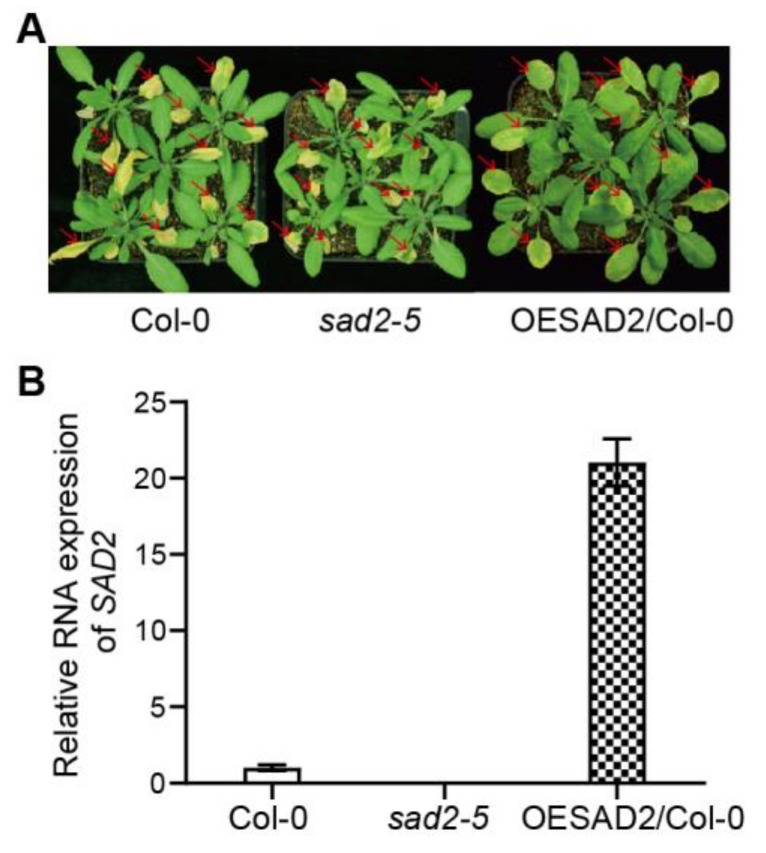
Disease symptoms and gene expression in Col-0, *SAD2* knockout (*sad2-5*), and *SAD2* overexpression (OESAD2/Col-0) leaves after inoculation with *Pseudomonas syringae* pv. tomato (*Pst*) DC3000. (**A**) Disease symptoms at four days post-inoculation with *Pst* DC3000. Red arrows indicate inoculated leaves. (**B**) *SAD2* expression in Col-0, *sad2-5*, and OESAD2/Col-0 plants.

In a previous study, we found that the nuclear transport protein SAD2 was involved in calcium and H_2_O_2_-mediated programmed cell death in Arabidopsis [23]. To investigate whether SAD2 was also involved in plant pathogen responses, we here measured pathogen susceptibility in Col-0, *sad2-5*, and OESAD2/Col-0 plants at 4 days post-inoculation (dpi) with *Pst* DC3000. The *sad2-5* mutant was more susceptible to *Pst* DC3000 infection than Col-0, whereas OESAD2/Col-0 was more resistant (Figure 1A). Quantitative reverse transcription RT-qPCR showed that *SAD2* was knocked out in *sad2-5* and expressed 21.03-fold higher in OESAD2/Col-0 than in Col-0 plants (Figure 1B). These results indicated that SAD2 may have a positive role in regulating the Arabidopsis pathogen response.

### 2.2. RNA-Sequencing Library Construction, Sequencing, and Read Mapping

To identify the genes regulated by *SAD2* in the pathogen resistance response, Col-0, *sad2-5*, and OESAD2/Col-0 leaves were collected at 0, 1, 2, and 3 dpi with *Pst* DC3000 and analyzed via RNA-sequencing (Table 1). The number of raw reads per sample ranged from 27,256,874 to 44,222,634, and the number of raw bases ranged from 4.08 to 6.63 Gb. We then performed quality control on the raw sequencing data, which included removing low-quality reads, deleting adapters, calculating the sequencing error rate, calculating the Q20 and Q30 values, and determining the GC content. The number of clean reads per sample ranged from 26,714,782 to 42,603,821, and the number of clean bases ranged from 4.01 to 6.39 Gb. The per-sample mapping rate to the reference genome ranged from 96.55 to 98.17%, and the total mapping rate showed that more than 96.55% of the clean reads were successfully aligned to the reference genome. Each species has a typical percentage of GC content, which can be compared to the GC content of a given sample to indicate whether there was interference from other species [33]. The GC content here ranged from 44.75% to 46.15%, demonstrating that the samples were relatively stable and did not have interference from other species. Base quality is an important indicator for measuring sequencing accuracy. Q20 and Q30 represent base misrecognition probabilities of 1% and 0.1%, respectively. The Q30 values for our data ranged from 93.36 to 94.58%, indicating high sequencing accuracy. Overall, the data quality assessments revealed that the sequencing data were of high quality and therefore suitable for further bioinformatics analyses.

### 2.3. DEGs in Col-0, sad2-5, and OESAD2/Col-0 after Pst DC3000 Infection

**Figure 2:**
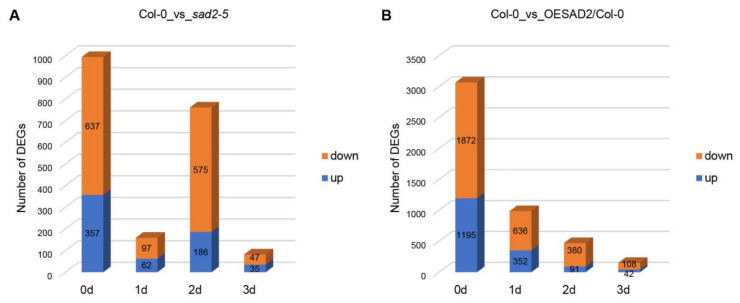
Differentially expressed genes (DEGs) in *SAD2* knockout and overexpression lines (*sad2-5* and OESAD2/Col-0, respectively) compared to Col-0. (**A**) DEGs between *sad2-5* and Col-0 at multiple time points after inoculation with *Pseudomonas syringae* pv. tomato (*Pst*) DC3000. (**B**) DEGs between OESAD2/Col-0 and Col-0 at multiple time points after inoculation with *Pst* DC3000. “d” here indicates days post-inoculation with *Pst* DC3000.

We next analyzed the sequencing data to identify DEGs in the *SAD2* knockout (*sad2-5)* and overexpression (OESAD2/Col-0) lines compared to Col-0 (Supplementary Table S1). At 0 dpi, there were a total of 994 DEGs in *sad2-5* compared to Col-0, with 357 up-regulated and 637 down-regulated genes (Figure 2A). There were 159 DEGs (62 up-regulated and 97 down-regulated) at 1 dpi, 1761 DEGs (186 up-regulated and 575 down-regulated) at 2 dpi, and 82 DEGs (35 up-regulated and 47 down-regulated) at 3 dpi (Figure 2A). In OESAD2/Col-0 samples, there were a total of 3067 DEGs at 0 dpi, with 1195 up-regulated and 1872 down-regulated (Figure 2B). There were 988 DEGs (352 up-regulated and 636 down-regulated) at 1 dpi, 471 DEGs (91 up-regulated and 380 down-regulated) at 2 dpi, and 150 DEGs (42 up-regulated and 108 down-regulated) at 3 dpi. The two mutant lines differed with respect to the dpi with the largest number of DEGs; in *sad2-5* compared to Col-0, it was at 2 dpi, whereas it was at 1 dpi for OESAD2/Col-0. Further analyses were then conducted on the DEGs.

### 2.4. Key DEGs Regulated by SAD2 and Involved in the Pst DC3000 Defense Response

**Figure 3:**
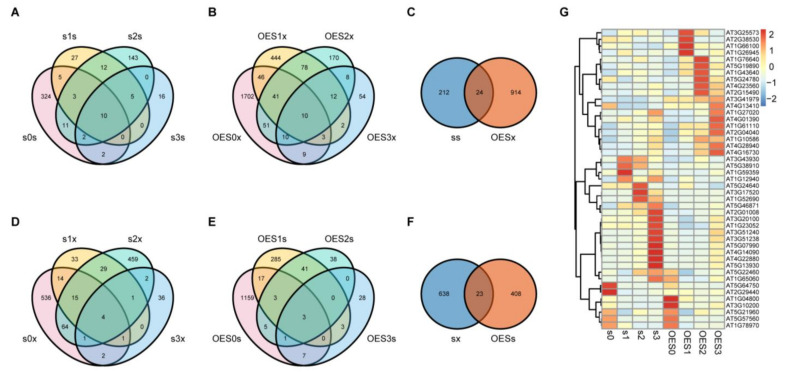
Venn and heatmap analyses of differentially expressed genes (DEGs). (**A**) Venn analysis of DEGs that were up-regulated in *sad2-5* compared with Col-0 at 0 days post-inoculation (dpi) (s0s), 1 dpi (s1s), 2 dpi (s2s), and 3 dpi (s3s) with *Pseudomonas syringae* pv. tomato (*Pst*) DC3000. (**B**) Venn analysis of DEGs that were down-regulated in the SAD2-overexpression line (OESAD2/Col-0) compared with Col-0 at 0 dpi (OES0x), 1 dpi (OES1x), 2 dpi (OES2x), and 3 dpi (OES3x). (**C**) Venn analysis of overlapping DEGs, i.e., those that were up-regulated in *sad2-5* (ss) and down-regulated in OESAD2/Col-0 (OESx). (**D**) Venn analysis of DEGs that were down-regulated in *sad2-5* compared to Col-0 at 0 dpi (s0x), 1 dpi (s1x), 2 dpi (s2x), and 3 dpi (s3x). (**E**) Venn analysis of DEGs that were up-regulated in OESAD2/Col-0 compared to Col-0 at 0 dpi (OES0s), 1 dpi (OES1s), 2 dpi (OES2s), and 3 dpi (OES3s). (**F**) Venn analysis of overlapping DEGs, i.e., those that were down-regulated in *sad2-5* (sx) and up-regulated in OESAD2/Col-0 (OESs). (**G**) Heatmap analysis of expression profiles of the 45 overlapping DEGs. “s” here indicates *sad2-5*; “OES” indicates OESAD2/Col-0; and numbers following “s” or “OES” indicate dpi with *Pst* DC3000.

A pathogen resistance test showed that *sad2-5* was more susceptible to *Pst* DC3000 infection than Col-0, whereas OESAD2/Col-0 exhibited resistance. This indicated that SAD2 positively regulated the pathogen defense response. Therefore, DEGs associated with pathogen resistance were expected to display contrasting expression profiles between *sad2-5* and OESAD2/Col-0 samples during *Pst* DC3000 infection. To identify the key DEGs that were regulated by *SAD2* and involved in response to *Pst* DC3000, we performed Venn and heatmap analyses (Figure 3).

In *sad2-5* plants, a total of 236 unique DEGs were up-regulated by *Pst* DC3000 over the three dpi (Figure 3A); in OESAD2/Col-0 plants, 938 DEGs were down-regulated by *Pst* DC3000 (Figure 3B). We then analyzed the genes that were common between these two sets, i.e., those that were up-regulated by *Pst* DC3000 infection in *sad2-5* but down-regulated in OESAD2/Col-0 (Figure 3C). This yielded a set of 24 genes in common, which were likely regulated by SAD2. Similarly, we analyzed the unique DEGs that were down-regulated by *Pst* DC3000 infection in *sad2-5* plants (661 DEGs) (Figure 3D), those that were up-regulated by *Pst* DC3000 infection in OESAD2/Col-0 plants (431 DEGs) (Figure 3E), and the overlap between those two sets (23 genes) (Figure 3F).

There were two genes in common between the 24 DEGs that were up-regulated in *sad2-5* but down-regulated in OESAD2/Col-0 and the 23 DEGs that were down-regulated in *sad2-5* but up-regulated in OESAD2/Col-0. Thus, we obtained a final set of 45 key common DEGs. Clustering the 45 DEGs based on relative expression levels in each genotype over the 3 dpi revealed three obvious expression patterns (Figure 3G; Supplementary Table S2). Nineteen DEGs displayed the first expression pattern, in which genes were significantly up-regulated in OESAD2/Col-0 after inoculation with *Pst* DC3000. For example, four DEGs (*AT3G25573*, *AT2G38530*, *AT1G66100*, and *AT1G26945*) were highly expressed in OESAD2/Col-0 but expressed at relatively low levels in *sad2-5* at 1 dpi. Six DEGS (*AT1G76640*, *AT5G19890*, *AT1G43640*, *AT5G24780*, *AT4G23560*, and *AT2G15490*) were highly expressed in OESAD2/Col-0 at 2 dpi, and nine DEGs (*AT3G41979*, *AT4G13410*, *AT1G27020*, *AT4G01390*, *AT1G61110*, *AT2G04040*, *AT1G10586*, *AT4G28940*, and *AT4G16730*) were highly expressed in OESAD2/Col-0 at 3 dpi. Another 19 DEGs displayed the second expression pattern, in which genes were up-regulated in *sad2-5* but down-regulated in OESAD2/Col-0 after *Pst* DC3000 inoculation. For example, *AT5G13930*, *AT4G22880*, and *AT5G07990* were highly expressed in *sad2-5* at 3 dpi but were expressed at relatively low levels in OESAD2/Col-0. Seven genes displayed the third expression pattern, in which there was weak induction by pathogen infection. In summary, after removing duplicate DEGs, we obtained a final set of 1825 DEGs that were induced by *Pst* DC3000 (Supplementary Figure S1), and 45 of those were key genes regulated by SAD2.

### 2.5. GO Term and KEGG Pathway Enrichment among Pathogen-Induced DEGs

**Figure 4:**
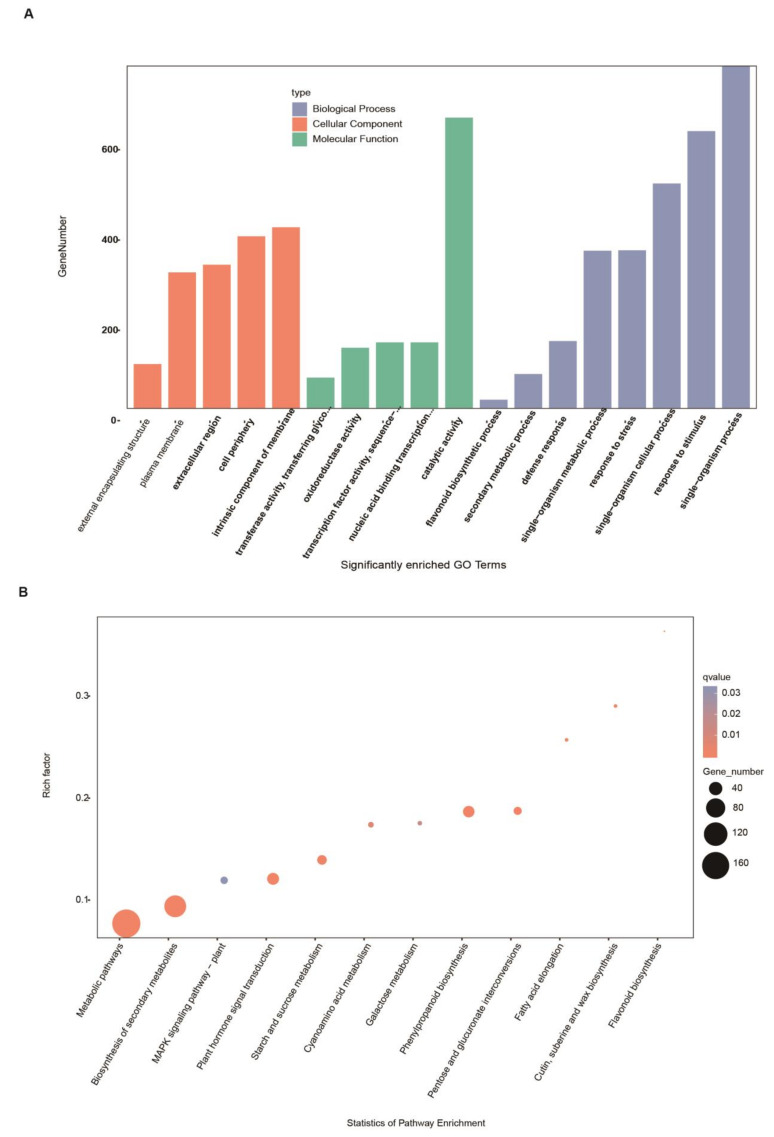
Gene Ontology (GO) and Kyoto Encyclopedia of Genes and Genomes (KEGG) biochemical pathway classifications of 1825 differentially expressed genes (DEGs). (**A**) Significantly enriched GO terms. (**B**) Significantly enriched KEGG biological pathway annotations. Annotations enriched among the 45 key SAD2-responsive DEGs are underlined in red.

Enriched Gene Ontology (GO) term analysis was next performed to determine functions that were enriched among DEGs (Figure 4A, Supplementary Table S3). Three types of GO annotations were analyzed: cellular components, molecular functions, and biological processes. Annotations were considered significantly enriched below the false discovery rate (FDR) threshold of 0.05. DEGs were mainly enriched in the biological process terms “single-organism process” (GO: 0044699, 760), “response to stimulus” (GO: 0050896, 615), “single-organism cellular process” (GO: 0044763, 499), “response to stress” (GO: 0006950, 351), and “single-organism metabolic process” (GO: 0044710350, 350). The most highly enriched molecular functions were “catalytic activity” (GO: 0003824, 645), “nucleic acid binding transcription factor activity” (GO: 0001071, 147), “transcription factor activity, sequence-specific DNA binding” (GO: 0003700, 147), “oxidoreductase activity” (GO: 0016491, 135), and “transferase activity, transferring glycosyl groups” (GO: 0016757, 69). The most highly enriched cellular component annotations were “intrinsic component of membrane” (GO: 0031224, 402), “cell periphery” (GO: 0071944), “extracellular region” (GO: 0005576, 382), “plasma membrane” (GO: 0005886, 302), and “external encapsulating structure” (GO: 0030312, 99). Importantly, the 45 key DEGs were mainly enriched in the terms “secondary metabolic process” (GO: 0019748, 7) and “flavonoid biosynthesis process” (GO: 0009813, 5).

We also performed Kyoto Encyclopedia of Genes and Genomes (KEGG) biochemical pathway enrichment analysis for the 1825 DEGs and 45 key DEGs using a threshold of Corrected *p*-Value ≤ 0.05 (Supplementary Table S4). The most highly enriched pathways included “metabolic pathways” (ath01100, 173), “biosynthesis of secondary metabolites” (ath01110, 104), “plant hormone signal transduction” (ath04075, 33), “phenylpropanoid biosynthesis” (ath00940, 31), and “starch and sucrose metabolism” (ath00500, 23) (Figure 4B). The 45 key DEGs were primarily enriched in the pathways “flavonoid biosynthesis” (ath00941, 4) and “biosynthesis of secondary metabolites” (ath01110, 7). Based on these results, we speculated that these pathways may have been related to SAD2-mediated regulation of the *Pst* DC3000-induced defense response.

### 2.6. Identification of Transcription Factors (TFs) Involved in the Pathogen Response

**Figure 5:**
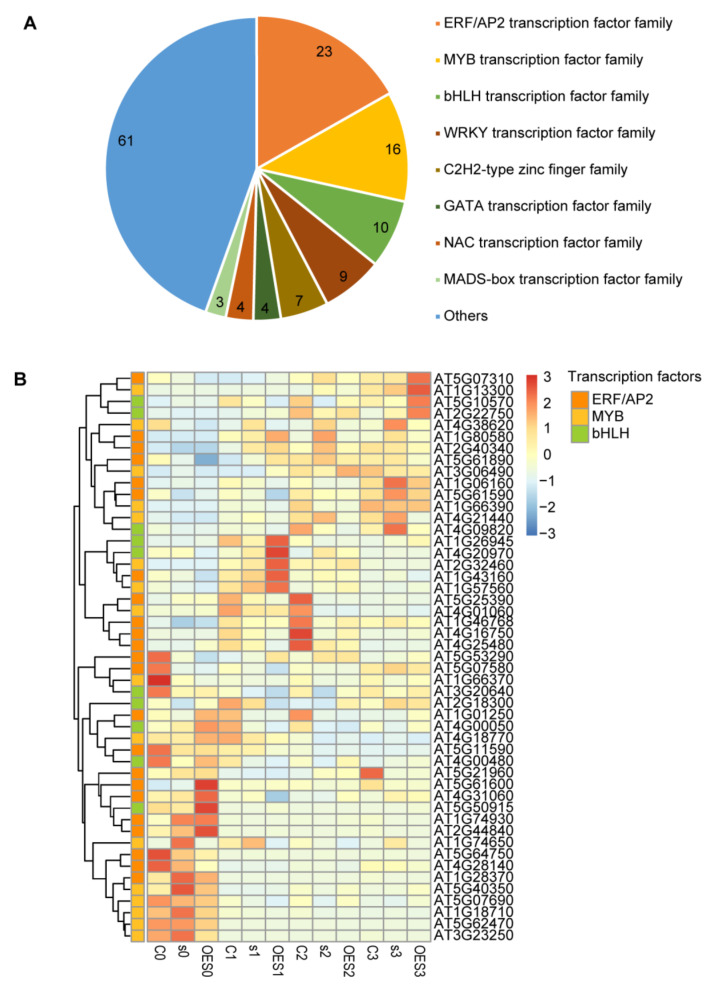
Expression levels of differentially expressed transcription factor (TF) family members in response to pathogen infection. (**A**) TF family classification statistics. (**B**) Heatmap showing expression levels of differentially expressed TFs in the ERF/AP2, MYB, and bHLH TF families in Col-0 (C), SAD2-knockout (s), and SAD2-overexpression (OES) plants. Numbers following “C”, “s”, or “OES” indicate days post-inoculation with *Pseudomonas syringae* pv. tomato DC3000.

TFs play important roles in many critical life activities in plants, including disease defense responses [34,35]. Most TFs must be transported to the nucleus to perform their functions. SAD2 belongs to a class of cytoplasmic transport receptor proteins and is responsible for cytoplasmic protein transport. We therefore focused on regulatory changes to TFs (i.e., TF expression levels) in response to SAD2 perturbation. Of the 1825 DEGs that we identified as responsive to *Pst* DC3000 infection, 137 DEGs were annotated as having TF activity; most of these belonged to eight main TF families: ERF/AP2, MYB, bHLH, WRKY, C2H2, GATA, NAC, and MADs-box (Figure 5A; Supplementary Table S5). The largest number of DEGs belonged to the ERF/AP2 TF family (23), followed by the MYB family (16) and the bHLH family (10). We next analyzed the expression levels of DEGs belonging to the ERF/AP2, MYB, and bHLH families in Col-0, *sad2-5*, and OESAD2/Col-0 plants (Figure 5B; Supplementary Table S6). Different expression patterns were observed for these genes between the three time points (1, 2, and 3 dpi). In total, 24 DEGs were up-regulated, and 25 were down-regulated after pathogen treatment. Notably, one set of genes that clustered together based on expression levels (*AT5G07310*, *AT1G13300*, *AT5G10570*, and *AT2G22750*) were significantly up-regulated in OESAD2/Col-0 plants at 3 dpi. Expression levels of genes in another cluster (*AT4G38620*, *AT1G80580*, *AT2G40340*, *AT5G61890*, *AT3G06490*, *AT1G06160*, *AT5G61590*, *AT1G66390*, *AT4G21440*, and *AT4G09820*) were significantly up-regulated in *sad2-5* plants at 3 dpi; the clustered genes *AT1G26945*, *AT4G20970*, *AT2G32460*, *AT1G43160*, and *AT1G57560* were significantly up-regulated in OESAD2/Col-0 plants at 1 dpi. The results suggested that these differentially expressed TFs may have been involved in the disease defense response mediated by the nucleocytoplasmic transport receptor SAD2 in Arabidopsis.

### 2.7. Verification of RNA-Seq Results via RT-qPCR

**Figure 6:**
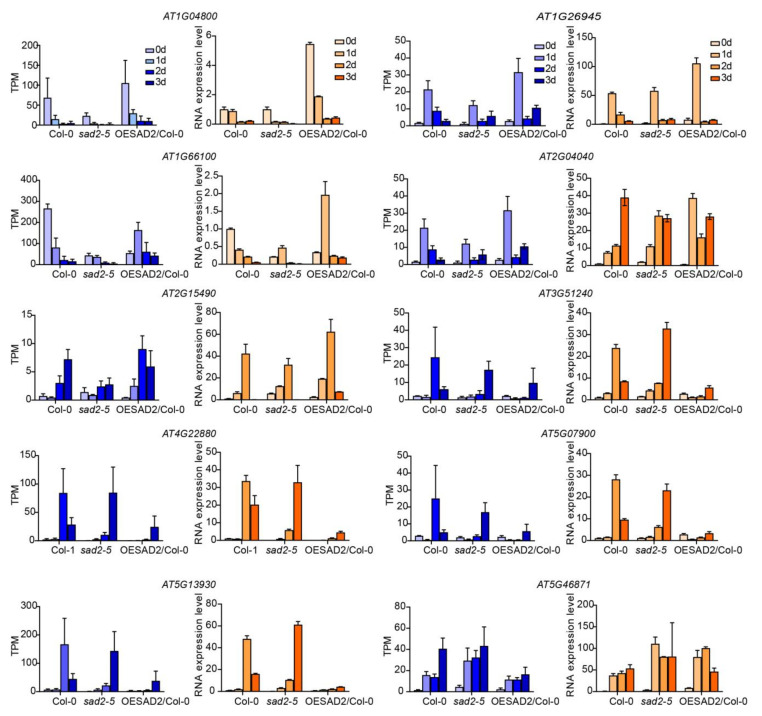
Verification of transcriptome sequencing results via RT-qPCR. The data were normalized by using *ACTIN1* (AT2G37620) as an internal reference. Data are represented as mean ± SD for three biological replicates. The TPM values of RNA-seq are shown in blue, and RT-qPCR results are shown in orange.

To confirm the reliability of the transcriptome data, we randomly selected 10 DEGs for verification with quantitative real-time polymerase chain reaction (RT-qPCR). The gene expression patterns observed in the RT-qPCR and RNA-seq data showed similar trends (Figure 6), indicating that the transcriptome sequencing results were reliable.

## 3. Discussion

We here studied Col-0 plants, th*e SA*D2 knockout mutant sad2-5, and the SAD2-overexpression lines OESAD2/Col-0 to identify defense response genes regulated by the nucleocytoplasmic transport receptor SAD2. At 4 dpi with *Pst* DC3000, *sad2-5* leaves were more susceptible than Col-0 leaves were, whereas OESAD2/Col-0 plants exhibited a distinct disease-resistant phenotype. Through transcriptomic analysis of inoculated leaves at 0, 1, 2, and 3 dpi, we identified 45 significant DEGs shared between *sad2-5* and OESAD2/Col-0 compared to Col-0 plants. These genes had contrasting expression patterns between *sad2-5* and OESAD2/Col-0 and were therefore classified as key candidate *SAD2*-mediated defense-related genes. For example, *AT2G38530*, also known as a lipid transfer protein 2 (LTP2), has been reported to play an important role in pathogen defense. Exogenous expression of barley *LTP2* in tobacco or Arabidopsis increases the tolerance of these plants to bacterial pathogens [36]. In the present study, the gene encoding LTP2 was significantly up-regulated in OESAD2/Col-0 plants (peaking at 1 dpi), whereas the expression level did not change in the susceptible genotype *sad2-5*. *AT4G28940*, a phosphorylase superfamily protein gene, was expressed at extremely low levels in *sad2-5* and OESAD2/Col-0 before *Pst* DC3000 inoculation; after *Pst* DC3000 inoculation, the gene was continuously up-regulated, and expression levels in OESAD2/Col-0 exceeded levels in *sad2-5* by ~1.5-fold. Previous studies have shown that F-type ATPase and α-1, 4 glucan phosphorylase may be important mediator proteins in the defense response to *Vibrio asiatica* [37]. The *Phytophthora soya* avirulent effector protein Avr3b is a secreted NADH and ADP-ribose pyrophosphorylase that regulates plant immunity [38]. Expression of a GDP-L-galactose phosphorylase-like gene in Chinese wild grape species induces responses to necrotrophic pathogens and defense signaling molecules [39]. We hypothesize that AT4G28940 (the phosphorylase superfamily protein) may be involved in anti-disease defense responses regulated by the nucleocytoplasmic transport receptor *SAD2*. AT2G04040 is a MATE efflux family protein. This gene was significantly up-regulated in OESAD2/Col-0, with the highest expression level at 3 dpi. MATE proteins have important roles in plant disease defense responses through both positive and negative regulatory mechanisms [40,41]. For example, the Arabidopsis gene *EDS5* encodes a MATE transporter homolog; an *eds5* mutant is highly susceptible to infection with several different pathogens, whereas *EDS5* overexpression in Arabidopsis increases pathogen resistance [42,43]. The MATE transporter DTX18 is a hydroxycinnamic acid amide (HCAA) coumaroylagmatine transporter with unique specificity that inhibits infection with Pseudomonas. In potato (*Solanum tuberosum*), DTX18 exports HCAAs to defend against the causal agent of late blight disease [44]. Furthermore, the MATE transporter *ADS1* negatively regulates the expression of salicylic acid (SA) biosynthetic genes and *PATHOGENESIS-RELATED GENE 1* (*PR-1*), increasing plant susceptibility to pathogens [45]. Likewise, overexpressing either one of two rice MATE transporter genes, *OsMATE1* or *OsMATE2*, in Arabidopsis increases susceptibility to *Pst* DC3000 [46]. Therefore, we speculate that these genes are involved in regulating pathogen defense responses.

The 45 putative *SAD2*-mediated defense-related DEGs were primarily enriched in the GO terms “secondary metabolic process” (GO:0019748, 7) and “flavonoid biosynthesis process” (GO:0009813, 5). GO annotation analysis revealed that SAD2 was involved in the regulation of disease resistance through single-organism cellular metabolic processes and responses to stress stimuli. The subcellular localization annotations associated with these processes were primarily the extracellular domain, plasma membrane, and intra-cellular membrane. Lipids are an integral part of cell membranes and play important roles in activating and mediating signal transduction pathways [47]. DEGs were also enriched in catalytic activity, nucleic-acid-binding TF activity, and oxidoreductase activity. *AT1G66100*, which was significantly up-regulated in OESAD2/Col-0, was annotated as a plant thionin that was mainly present in the extracellular region (GO: 0005576) and was involved in biological processes such as “response to stimulus” (GO: 0050896). Thionin is hypothesized to induce pore formation in pathogenic cell membranes, allowing potassium and calcium ion leakage [48,49]. Numerous studies have shown that the secreted antifungal compound thionin ASTHI2.4 inhibits the toxicity of fungal fruiting body lectins in *Fusarium graminearum* [50]. In citrus, overexpression of a modified plant thionin enhances disease resistance to citrus canker and Huanglongbing (HLB) [51]. Transgenic sweet potatoes overexpressing thionin are resistant to black rot caused by *Ceratocystis fimbriata* in the leaves and storage roots [52]. Therefore, we posit that *AT1G66100* (a plant thionin) was involved in the bacterial defense response regulated by *SAD2*.

KEGG analysis revealed that the most highly enriched biochemical pathways among the DEGs were “metabolic pathways” and “biosynthesis of secondary metabolites”. In addition, the 45 putative key DEGs were significantly enriched in “biosynthesis of secondary metabolites” and “flavonoid biosynthesis”, consistent with the GO term analysis. Flavonoids are a class of plant-specialized metabolites (PSMs) that protect against pathogen infection [53]. PSMs have important functions in mediating interactions between plants and other organisms. Metabolites produced by the phenylpropane metabolic pathway (e.g., flavonoids, phenols, and lignins) are also involved in plant pathogen defense responses [54]. Flavanone 3-hydroxylase (F3H) is a key player in the flavonoid biosynthetic pathway, which can coordinate plant defense mechanisms and enhance plant tolerance to biotic and abiotic stresses. A previous study showed that excessive flavonoid accumulation can enhance ROS scavenging in Arabidopsis, improving plant antioxidant activity and drought resistance [55]. Rice lines overexpressing F3H have enhanced tolerance to bacterial leaf blight (BLB) compared to wild-type plants due to excessive accumulation of antioxidant flavonoids [56]. In addition, several members of the flavonoid biosynthetic pathway were differentially expressed in *sad2-5* and OESAD2/Col-0 compared to Col-0, namely AT4G22880 (leucoanthocyanidin dioxygenase [LDOX]), AT5G13930 (TT4, a chalcone and stilbene synthase family protein), and AT5G07990 (TT7, a cytochrome P450 superfamily protein). This pathway is also closely related to plant disease resistance responses. SA, jasmonate (JA), and ethylene (ET) are important components of plant defense signaling [57]. Pathogen-triggered biosynthesis of these defense hormones and their subsequent signaling activity induces activation of a complex set of defense-related genes, leading to multiple defense responses. The DEGs identified here as related to disease resistance, and defense responses were significantly enriched in MAPK signaling [58], starch and sucrose metabolism, and fatty acid elongation pathways. These results suggest that the flavonoid and specialized metabolite pathways have important roles in SAD2-mediated disease resistance.

TFs are important factors in biotic stress responses, such as those induced by insect pests or pathogens. Numerous studies have reported the involvement of many TF families in plant pathogen defense responses. For example, the ET response factor ERF11 enhances plant resistance to bacterial pathogens [59]. The ERF TF GmERF113 increases soybean resistance to *Phytophthora soja* [60]. The tomato ERF TF SlERF84 enhances tolerance to drought and salt stress but negatively regulates defense responses to *Pst* DC3000 [61]. Overexpression of the apple (*Malus domestica*) gene *MdERF100* in Arabidopsis enhances resistance to powdery mildew by regulating the JA and SA signaling pathways [62]. R2R3-MYB TFs can improve brown planthopper resistance in rice by regulating the phenylalanine ammonia lyase pathway [63]. In wheat (*Triticum aestivum*), the TF TaMYB29 is involved in the prevention of wheat stripe rust and is significantly induced by SA, abscisic acid (ABA), JA, ET, and *Pst* [64]. In rice (*Oryza sativa*), a bHLH transcriptional activator, OsbHLH6, has been identified as an enhancer of disease resistance by dynamically regulating SA and JA signaling through nuclear-cytoplasmic trafficking [65]. MdbHLH92 is involved in plant defense against powdery mildew [62]. In addition, knocking down *bHLH132* in tomato stunts plant development and enhances susceptibility to *Xanthomonas euvesicatoria* [66]. We here identified a total of 137 DEGs with TF activity that may have been involved in SAD2-mediated defense responses; most such TFs belonged to the EFR, MYB, and bHLH families (23, 10, and 9 DEGs, respectively). Two of these genes (*AT1G26945* and *AT1G10586*) were bHLH DNA-binding superfamily proteins and were specifically up-regulated in OESAD2/Col-0 plants at 1 and 3 dpi. We hypothesize that they may participate in defense responses that are regulated by the nuclear cytoplasmic transport receptor SAD2.

In summary, we here analyzed RNA-sequencing data from wild-type (Col-0), SAD2 knockout mutant (*sad2-5*), and SAD2-overexpression (OESAD2/Col-0) plants. Each genotype was inoculated with *Pst* DC3000 and analyzed at multiple time points. The resulting gene expression profiles revealed key candidate genes and metabolic pathways that appeared to be involved in defense responses that are regulated by the nucleocytoplasmic transport receptor SAD2. These results improve our understanding of the mechanisms by which nuclear cytoplasmic transport receptors mediate plant defense responses. Furthermore, this study provides a foundation for future exploration of the molecular mechanisms associated with SAD2-mediated disease resistance and establishes a set of strong candidate plant disease-resistance genes

## 4. Materials and Methods

### 4.1. Plant Materials and Growth Conditions

Arabidopsis ecotype Col-0, the SAD2 knockout mutant *sad2-5*, and the SAD2-overexpression line OESAD2/Col-0 were used in this study. *sad2-5* is a previously described T-DNA insertion line [29,30]. OESAD2/Col-0 was generated for the present study by inserting the coding region of SAD2 into the pCAMBIA1307 vector under the control of the cauliflower mosaic virus 35S promoter. Col-0 was transformed with the recombinant plasmid by Agrobacterium-mediated transformation via floral dip, and T4 homozygous lines were used for this study.

For plants used in susceptibility analyses and RNA-seq assays, seeds were sterilized and sown on 1/2× Murashige and Skoog (MS) medium containing 2.5% (*w*/*v*) sucrose and 1% (*w*/*v*) agar. At approximately 1 week of age, seedlings were transplanted into soil and raised in a growth chamber at 22 °C with a 16/8 h light/dark cycle at 60–70% relative humidity.

### 4.2. Arabidopsis Inoculation with Pst DC3000 and Leaf Sample Collection

*Pst* DC3000 was cultured on King’s B (KB) solid medium with 25 µg/mL rifampicin at 28 °C for 2 d. Single colonies were cultured in KB liquid medium overnight at 28 °C with shaking, reaching an OD600 ≥ 1. The OD600 was then adjusted to 0.002 with sterile ddH_2_O. Arabidopsis rosette leaves were infiltrated with the bacterial suspension using 1 mL needleless syringes. Leaves were collected in biological triplicate at 0, 1, 2, and 3 dpi, immediately frozen in liquid nitrogen, and stored at −80 °C prior to RNA extraction.

### 4.3. RNA Extraction, cDNA Library Construction, and Illumina Deep Sequencing

Total RNA was extracted from leaf samples using TRIzol reagent (Invitrogen, Thermo Fisher Scientific, Beijing, China) following the manufacturer’s protocol. The purity, concentration, and quality of RNA samples were measured using a NanoDrop 2000 spectrophotometer (NanoDrop, Wilmington, DE, USA) to ensure sample suitability. RNA quality was also verified through visualization of ~500 ng RNA per sample on a 1% agarose gel. From suitably high-quality RNA samples, library construction and Illumina sequencing were completed at Allwegene Technology (Beijing, China). The HiSeq/NovaSeq PE150 platform was used and resulted in ~6 Gb of clean data per sample.

### 4.4. RNA-Sequencing Data Analysis

Raw reads obtained from the sequencing facility included adaptor sequences and low-quality sequences. To ensure the accuracy of downstream analyses, we used fastp [67] to clean raw reads and FASTQC (http://www.bioinformatics.babraham.ac.uk/projects/fastqc, accessed on 20 October 2020) to evaluate the quality. Clean reads were aligned to the TAIR10 Arabidopsis reference genome with Hisat2 [68]. After alignment, transcript abundance was quantified for each gene with SAMtools (http://samtools.sourceforge.net/, accessed on 25 December 2020) [69] and HTSeq (http://www-huber.embl.de/HTSeq/doc/overview.html, accessed on 8 March 2021) [70]. The Bioconductor ‘DESeq2’ [71] package in R was used to identify DEGs between genotypes at each time point. For gene expression analysis, the number of unambiguous tags for each gene was calculated and normalized to the number of transcripts per million tags (TPM). Genes with FDR ≤ 0.05 and |log2(FC)| ≥ 1 were classified as significant DEGs.

### 4.5. Gene Enrichment Analysis

TBtools [72] was used to generate Venn diagrams to display unique and overlapping DEGs between groups. The ‘heatmap’ package in R was used to draw heatmaps. To determine the biological functions of DEGs, we used the online tool AgriGO (http://systemsbiology.cau.edu.cn/agriGOv2/index.php, accessed on 10 October 2021) to perform GO enrichment analysis [73]. GO terms with FDR ≤ 0.05 were considered significantly enriched. For biochemical pathway analysis, we used DAVID (https://david.ncifcrf.gov/home.jsp, accessed on 16 November 2021) to convert each gene ID to the corresponding Entrez gene ID, then entered those gene IDs into the KOBAS website (http://kobas.cbi.pku.edu.cn/kobas3/genelist/, accessed on 16 November 2021). KEGG biochemical pathway enrichment analysis was conducted for DEGs using the KEGG database [74], with *p* ≤ 0.05 used as the threshold for classifying significantly enriched pathways.

### 4.6. Validation of RNA-Seq Data

The primers used for RT-qPCR (Supplementary Table S7) were designed with Primer-BLAST (https://www.ncbi.nlm.nih.gov/tools/primer-blast/, accessed on 23 October 2022) and synthesized by Sangon Biotech (Shanghai, China). *ACTIN1* (*AT2G37620*) was used as the internal reference gene to normalize expression levels using the 2^−ΔΔCt^ method [75]. Approximately 2 μg of total RNA was used for first-strand cDNA synthesis (Tiangen Biotech, Beijing, China). RT-qPCR was performed in 20 μL reaction volumes using SYBR Premix Ex TaqTM II (TAKARA BIO INC., Shiga, Japan) on a 7500 Fast Real-Time PCR System (Applied Biosystems, Foster City, CA, USA) with the following conditions: 95 °C for 30 s, then 40 cycles of 95 °C for 5 s and 60 °C for 34 s.

## Figures and Tables

**Table 1:** Summary of sequencing results.

Samples	Replicate	Raw Reads	Raw Bases	Clean Reads	Clean Bases	Q20 (%)	Q30 (%)	GC (%)	Total_ Mapping (%)
Col-0_0d	1	35,015,991	5.25 G	34,168,202	5.13 G	98.01	94.20	45.34	97.79
	2	35,996,956	5.39 G	34,874,632	5.23 G	97.97	94.14	45.48	98.02
	3	39,606,195	5.94 G	38,314,248	5.75 G	98.06	94.37	45.27	97.93
Col-0_1d	1	39,189,239	5.87 G	38,014,575	5.7 G	98.07	94.38	44.98	97.98
	2	38,505,552	5.77 G	37,168,785	5.58 G	98.07	94.35	45.05	98.05
	3	40,696,320	6.1 G	39,371,453	5.91 G	97.95	94.12	45.25	97.90
Col-0_2d	1	38,202,509	5.73 G	37,115,329	5.57 G	97.98	94.12	44.75	98.03
	2	39,131,200	5.86 G	37,617,017	5.64 G	97.96	94.09	45.11	98.00
	3	42,585,194	6.38 G	41,201,001	6.18 G	97.98	94.17	45.11	97.06
Col-0_3d	1	38,249,317	5.73 G	37,029,249	5.55 G	98.09	94.41	44.87	98.04
	2	35,949,275	5.39 G	34,517,786	5.18 G	98.02	94.22	45.01	97.92
	3	34,922,101	5.23 G	33,703,571	5.06 G	98.14	94.54	44.98	98.08
sad2-5_0d	1	37,867,490	5.68 G	36,537,734	5.48 G	98.11	94.48	45.32	97.33
	2	40,558,326	6.08 G	39,307,108	5.9 G	98.08	94.43	45.26	97.29
	3	44,222,634	6.63 G	42,603,821	6.39 G	98.06	94.44	45.37	96.85
sad2-5_1d	1	38,518,272	5.77 G	37,239,684	5.59 G	98.11	94.48	45.13	97.60
	2	39,760,691	5.96 G	38,544,874	5.78 G	97.98	94.12	45.05	97.60
	3	38,946,660	5.84 G	37,622,323	5.64 G	98.00	94.18	45.00	97.37
sad2-5_2d	1	35,927,275	5.38 G	34,755,848	5.21 G	98.16	94.58	44.97	97.56
	2	38,878,184	5.83 G	37,212,316	5.58 G	97.73	93.36	45.23	97.34
	3	36,917,936	5.53 G	35,742,305	5.36 G	97.99	94.20	45.10	97.27
sad2-5_3d	1	34,058,842	5.1 G	32,974,801	4.95 G	98.07	94.38	45.16	97.47
	2	38,813,455	5.82 G	37,614,495	5.64 G	98.12	94.45	44.95	97.34
	3	41,067,633	6.16 G	40,173,877	6.03 G	97.79	93.66	45.37	96.55
OESAD2_0d	1	33,942,065	5.09 G	33,246,869	4.99 G	98.03	94.25	45.89	98.06
	2	30,279,425	4.54 G	29,684,633	4.45 G	98.09	94.36	45.80	98.17
	3	39,884,234	5.98 G	38,968,579	5.85 G	98.10	94.43	46.15	98.11
OESAD2_1d	1	38,695,180	5.8 G	37,829,383	5.67 G	97.83	93.78	45.30	97.81
	2	41,162,035	6.17 G	40,342,339	6.05 G	98.03	94.23	45.24	98.05
	3	27,256,874	4.08 G	26,714,782	4.01 G	97.90	93.90	45.18	98.00
OESAD2_2d	1	32,949,733	4.94 G	32,275,938	4.84 G	97.91	93.95	45.19	97.85
	2	41,170,080	6.17 G	40,515,870	6.08 G	98.05	94.28	45.34	97.57
	3	35,475,771	5.32 G	34,878,026	5.23 G	98.12	94.42	45.18	98.03
OESAD2_3d	1	39,301,446	5.89 G	38,509,059	5.78 G	97.87	93.82	45.15	96.70
	2	40,545,374	6.08 G	39,523,760	5.93 G	97.97	94.14	45.36	97.15
	3	40,018,743	6 G	39,135,547	5.87 G	98.01	94.19	45.34	97.86

## Data Availability

The raw sequence data reported in this paper have been deposited in the Genome Sequence Archive (Genomics, Proteomics & Bioinformatics 2021) at National Genomics Data Center (Nucleic Acids Res 2022), China National Center for Bioinformation/Beijing Institute of Genomics, Chinese Academy of Sciences (GSA: CRA009197), publicly accessible at https://ngdc.cncb.ac.cn/gsa, accessed on 15 December 2022.

## Supplementary Materials

The supporting information can be downloaded at: https://www.mdpi.com/article/10.3390/ijms24044229/s1.

## References

[1] Savary S., Willocquet L., Pethybridge S.J., Esker P., McRoberts N., Nelson A. (2019). The global burden of pathogens and pests on major food crops. Nat. Ecol. Evol..

[2] Bailey-Serres J., Parker J.E., Ainsworth E.A., Oldroyd G.E.D., Schroeder J.I. (2019). Genetic strategies for improving crop yields. Nature.

[3] Boyd L.A., Ridout C., O’Sullivan D.M., Leach J.E., Leung H. (2013). Plant-pathogen interactions: Disease resistance in modern agriculture. Trends Genet..

[4] Yan W., Ni Y., Liu X., Zhao H., Chen Y., Jia M., Liu M., Liu H., Tian B. (2021). The mechanism of sesame resistance against Macrophomina phaseolina was revealed via a comparison of transcriptomes of resistant and susceptible sesame genotypes. BMC Plant. Biol..

[5] Islam M.T., Hussain H.I., Rookes J.E., Cahill D.M. (2018). Transcriptome analysis, using RNA-Seq of Lomandra longifolia roots infected with Phytophthora cinnamomi reveals the complexity of the resistance response. Plant. Biol..

[6] Wang W., Feng B., Zhou J.M., Tang D. (2020). Plant immune signaling: Advancing on two frontiers. J. Integr. Plant. Biol..

[7] Saijo Y., Loo E.P. (2020). Plant immunity in signal integration between biotic and abiotic stress responses. New Phytol..

[8] Meng X., Zhang S. (2013). MAPK cascades in plant disease resistance signaling. Annu. Rev. Phytopathol..

[9] Zhou J.M., Zhang Y. (2020). Plant Immunity: Danger Perception and Signaling. Cell.

[10] Shi T., Gao Y., Wang H., Liu J. (2021). Nucleo-cytoplasmic Transport and Transport Receptors in Plant Disease Resistance Defense Response. Chin. Bull. Bot..

[11] Schwessinger B., Ronald P.C. (2012). Plant innate immunity: Perception of conserved microbial signatures. Ann. Rev. Plant. Biol..

[12] Boller T., He S.Y. (2009). Innate Immunity in Plants: An Arms Race Between Pattern Recognition Receptors in Plants and Effectors in Microbial Pathogens. Science.

[13] Feng F., Zhou J.M. (2012). Plant-bacterial pathogen interactions mediated by type III effectors. Curr. Opin. Plant. Biol..

[14] Dodds P.N., Rathjen J.P. (2010). Plant immunity: Towards an integrated view of plant-pathogen interactions. Nat. Rev. Genet..

[15] Nishimura M.T., Dangl J.L. (2010). Arabidopsis and the plant immune system. Plant. J..

[16] Jacob F., Kracher B., Mine A., Seyfferth C., Blanvillain-Baufume S., Parker J.E., Tsuda K., Schulze-Lefert P., Maekawa T. (2018). A dominant-interfering camta3 mutation compromises primary transcriptional outputs mediated by both cell surface and intracellular immune receptors in Arabidopsis thaliana. New Phytol..

[17] Lolle S., Stevens D., Coaker G. (2020). Plant NLR-triggered immunity: From receptor activation to downstream signaling. Curr. Opin. Immunol..

[18] Zhang Y.L., Li X. (2005). A putative nucleoporin 96 is required for both basal defense and constitutive resistance responses mediated by suppressor of npr1-1, constitutive 1. Plant. Cell.

[19] Liu J., Coaker G. (2008). Nuclear trafficking during plant innate immunity. Molecular. Plant.

[20] Park C.J., Ronald P.C. (2012). Cleavage and nuclear localization of the rice XA21 immune receptor. Nat. Commun..

[21] Deslandes L., Rivas S. (2011). The plant cell nucleus: A true arena for the fight between plants and pathogens. Plant. Signal. Behav..

[22] Wiermer M., Palma K., Zhang Y., Li X. (2007). Should I stay or should I go? Nucleocytoplasmic trafficking in plant innate immunity. Cell Microbiol..

[23] Teh O.K., Hofius D. (2014). Membrane trafficking and autophagy in pathogen-triggered cell death and immunity. J. Exp. Bot..

[24] Gu Y., Zebell S.G., Liang Z., Wang S., Kang B.H., Dong X. (2016). Nuclear Pore Permeabilization Is a Convergent Signaling Event in Effector-Triggered Immunity. Cell.

[25] Wang W.M., Liu P.Q., Xu Y.J., Xiao S. (2016). Protein trafficking during plant innate immunity. J. Integr. Plant. Biol..

[26] Xu S., Zhang Z., Jing B., Gannon P., Ding J., Xu F., Li X., Zhang Y. (2011). Transportin-SR is required for proper splicing of resistance genes and plant immunity. PLoS Genet..

[27] Xu K., Tao T., Jie J., Lu X.D., Li X.Z., Mehmood M.A., He H., Liu Z., Xiao X.Y., Yang J. (2013). Increased importin 13 activity is associated with the pathogenesis of pterygium. Mol. Vis..

[28] Kimura M., Imamoto N. (2014). Biological significance of the importin-beta family-dependent nucleocytoplasmic transport pathways. Traffic.

[29] Verslues P.E., Guo Y., Dong C.H., Ma W., Zhu J.K. (2006). Mutation of SAD2, an importin beta-domain protein in Arabidopsis, alters abscisic acid sensitivity. Plant. J..

[30] Zhao J., Zhang W., Zhao Y., Gong X., Guo L., Zhu G., Wang X., Gong Z., Schumaker K.S., Guo Y. (2007). SAD2, an importin -like protein, is required for UV-B response in Arabidopsis by mediating MYB4 nuclear trafficking. Plant. Cell.

[31] Gao Y., Gong X., Cao W., Zhao J., Fu L., Wang X., Schumaker K.S., Guo Y. (2008). SAD2 in Arabidopsis functions in trichome initiation through mediating GL3 function and regulating GL1, TTG1 and GL2 expression. J. Integr. Plant. Biol..

[32] Zheng Y., Zhan Q., Shi T., Liu J., Zhao K., Gao Y. (2020). The nuclear transporter SAD2 plays a role in calcium- and H2 O2 -mediated cell death in Arabidopsis. Plant. J..

[33] Smarda P., Bures P., Horova L., Leitch I.J., Mucina L., Pacini E., Tichy L., Grulich V., Rotreklova O. (2014). Ecological and evolutionary significance of genomic GC content diversity in monocots. Proc. Natl. Acad. Sci. USA.

[34] Ng D.W., Abeysinghe J.K., Kamali M. (2018). Regulating the Regulators: The Control of Transcription Factors in Plant Defense Signaling. Int. J. Mol. Sci..

[35] Amorim L.L.B., da Fonseca Dos Santos R., Neto J.P.B., Guida-Santos M., Crovella S., Benko-Iseppon A.M. (2017). Transcription Factors Involved in Plant Resistance to Pathogens. Curr. Protein Pept. Sci..

[36] Molina A., García-Olmedo F. (2003). Enhanced tolerance to bacterial pathogens caused by the transgenic expression of barley lipid transfer protein LTP2. Plant J..

[37] Li B., Zhang Y., Qiu D., Francis F., Wang S. (2021). Comparative Proteomic Analysis of Sweet Orange Petiole Provides Insights Into the Development of Huanglongbing Symptoms. Front. Plant. Sci..

[38] Dong S., Yin W., Kong G., Yang X., Qutob D., Chen Q., Kale S.D., Sui Y., Zhang Z., Dou D. (2011). Phytophthora sojae avirulence effector Avr3b is a secreted NADH and ADP-ribose pyrophosphorylase that modulates plant immunity. PLoS Pathog..

[39] Hou H.M., Li H.E., Gao M., Wang H., Jiao C., Wang X.P. (2013). Expression of a GDP-L-galactose phosphorylase-like gene in a Chinese wild Vitis species induces responses to Erysiphe necator and defense signaling molecules. Genet. Mol. Res..

[40] Upadhyay N., Kar D., Deepak Mahajan B., Nanda S., Rahiman R., Panchakshari N., Bhagavatula L., Datta S. (2019). The multitasking abilities of MATE transporters in plants. J. Exp. Bot..

[41] Devanna B.N., Jaswal R., Singh P.K., Kapoor R., Jain P., Kumar G., Sharma Y., Samantaray S., Sharma T.R. (2021). Role of transporters in plant disease resistance. Physiol. Plant..

[42] Nawrath C., Heck S., Parinthawong N., Metraux J.P. (2002). EDS5, an essential component of salicylic acid-dependent signaling for disease resistance in Arabidopsis, is a member of the MATE transporter family. Plant. Cell.

[43] Ishihara T., Sekine K.T., Hase S., Kanayama Y., Seo S., Ohashi Y., Kusano T., Shibata D., Shah J., Takahashi H. (2008). Overexpression of the Arabidopsis thaliana EDS5 gene enhances resistance to viruses. Plant. Biol..

[44] Dobritzsch M., Lubken T., Eschen-Lippold L., Gorzolka K., Blum E., Matern A., Marillonnet S., Bottcher C., Drager B., Rosahl S. (2016). MATE Transporter-Dependent Export of Hydroxycinnamic Acid Amides. Plant. Cell.

[45] Sun X., Gilroy E.M., Chini A., Nurmberg P.L., Hein I., Lacomme C., Birch P.R., Hussain A., Yun B.W., Loake G.J. (2011). ADS1 encodes a MATE-transporter that negatively regulates plant disease resistance. New Phytol..

[46] Tiwari M., Sharma D., Singh M., Tripathi R.D., Trivedi P.K. (2014). Expression of OsMATE1 and OsMATE2 alters development, stress responses and pathogen susceptibility in Arabidopsis. Sci. Rep..

[47] Shah J. (2005). Lipids, lipases, and lipid-modifying enzymes in plant disease resistance. Annu. Rev. Phytopathol..

[48] Pelegrini P.B., Franco O.L. (2005). Plant gamma-thionins: Novel insights on the mechanism of action of a multi-functional class of defense proteins. Int. J. Biochem. Cell Biol..

[49] Oard S.V. (2011). Deciphering a mechanism of membrane permeabilization by alpha-hordothionin peptide. Biochim. Biophys. Acta.

[50] Asano T., Miwa A., Maeda K., Kimura M., Nishiuchi T. (2013). The secreted antifungal protein thionin 2.4 in Arabidopsis thaliana suppresses the toxicity of a fungal fruit body lectin from Fusarium graminearum. PLoS Pathog..

[51] Hao G., Stover E., Gupta G. (2016). Overexpression of a Modified Plant Thionin Enhances Disease Resistance to Citrus Canker and Huanglongbing (HLB). Front. Plant. Sci..

[52] Muramoto N., Tanaka T., Shimamura T., Mitsukawa N., Hori E., Koda K., Otani M., Hirai M., Nakamura K., Imaeda T. (2012). Transgenic sweet potato expressing thionin from barley gives resistance to black rot disease caused by Ceratocystis fimbriata in leaves and storage roots. Plant. Cell Rep..

[53] Falcone Ferreyra M.L., Rius S.P., Casati P. (2012). Flavonoids: Biosynthesis, biological functions, and biotechnological applications. Front. Plant. Sci..

[54] Le Roy J., Huss B., Creach A., Hawkins S., Neutelings G. (2016). Glycosylation Is a Major Regulator of Phenylpropanoid Availability and Biological Activity in Plants. Front. Plant. Sci..

[55] Nakabayashi R., Yonekura-Sakakibara K., Urano K., Suzuki M., Yamada Y., Nishizawa T., Matsuda F., Kojima M., Sakakibara H., Shinozaki K. (2014). Enhancement of oxidative and drought tolerance in Arabidopsis by overaccumulation of antioxidant flavonoids. Plant. J..

[56] Jan R., Aaqil Khan M., Asaf S., Lubna, Park J.R., Lee I.J., Kim K.M. (2021). Flavonone 3-hydroxylase Relieves Bacterial Leaf Blight Stress in Rice via Overaccumulation of Antioxidant Flavonoids and Induction of Defense Genes and Hormones. Int. J. Mol. Sci..

[57] Bari R., Jones J.D. (2009). Role of plant hormones in plant defence responses. Plant. Mol. Biol..

[58] Wang X., Cheng C., Li Q., Zhang K., Lou Q., Li J., Chen J. (2020). Multi-omics analysis revealed that MAPK signaling and flavonoid metabolic pathway contributed to resistance against Meloidogyne incognita in the introgression line cucumber. J. Proteomics.

[59] Wu J., Deng Y., Hu J., Jin C., Zhu X., Li D. (2020). Genome-wide analyses of direct target genes of an ERF11 transcription factor involved in plant defense against bacterial pathogens. Biochem. Biophys Res. Commun..

[60] Zhao Y., Chang X., Qi D., Dong L., Wang G., Fan S., Jiang L., Cheng Q., Chen X., Han D. (2017). A Novel Soybean ERF Transcription Factor, GmERF113, Increases Resistance to Phytophthora sojae Infection in Soybean. Front. Plant. Sci..

[61] Li Z., Tian Y., Xu J., Fu X., Gao J., Wang B., Han H., Wang L., Peng R., Yao Q. (2018). A tomato ERF transcription factor, SlERF84, confers enhanced tolerance to drought and salt stress but negatively regulates immunity against Pseudomonas syringae pv. tomato DC3000. Plant. Physiol. Biochem..

[62] Zhang Y., Zhang L., Ma H., Zhang Y., Zhang X., Ji M., van Nocker S., Ahmad B., Zhao Z., Wang X. (2021). Overexpression of the Apple (Malus x domestica) MdERF100 in Arabidopsis Increases Resistance to Powdery Mildew. Int. J. Mol. Sci..

[63] He J., Liu Y., Yuan D., Duan M., Liu Y., Shen Z., Yang C., Qiu Z., Liu D., Wen P. (2020). An R2R3 MYB transcription factor confers brown planthopper resistance by regulating the phenylalanine ammonia-lyase pathway in rice. Proc. Natl. Acad. Sci. USA.

[64] Zhu X., Li X., He Q., Guo D., Liu C., Cao J., Wu Z., Kang Z., Wang X. (2021). TaMYB29: A Novel R2R3-MYB Transcription Factor Involved in Wheat Defense Against Stripe Rust. Front. Plant. Sci..

[65] Meng F., Yang C., Cao J., Chen H., Pang J., Zhao Q., Wang Z., Qing Fu Z., Liu J. (2020). A bHLH transcription activator regulates defense signaling by nucleo-cytosolic trafficking in rice. J. Integr. Plant. Biol..

[66] Kim J.G., Mudgett M.B. (2019). Tomato bHLH132 Transcription Factor Controls Growth and Defense and Is Activated by Xanthomonas euvesicatoria Effector XopD During Pathogenesis. Mol. Plant. Microbe Interact..

[67] Chen S., Zhou Y., Chen Y., Gu J. (2018). fastp: An ultra-fast all-in-one FASTQ preprocessor. Bioinformatics.

[68] Kim D., Langmead B., Salzberg S.L. (2015). HISAT: A fast spliced aligner with low memory requirements. Nat. Methods.

[69] Li H., Handsaker B., Wysoker A., Fennell T., Ruan J., Homer N., Marth G., Abecasis G., Durbin R., Genome Project Data Processing S. (2009). The Sequence Alignment/Map format and SAMtools. Bioinformatics.

[70] Anders S., Pyl P.T., Huber W. (2015). HTSeq--a Python framework to work with high-throughput sequencing data. Bioinformatics.

[71] Love M.I., Huber W., Anders S. (2014). Moderated estimation of fold change and dispersion for RNA-seq data with DESeq2. Genome Biol..

[72] Chen C., Chen H., Zhang Y., Thomas H.R., Frank M.H., He Y., Xia R. (2020). TBtools: An Integrative Toolkit Developed for Interactive Analyses of Big Biological Data. Mol. Plant.

[73] Tian T., Liu Y., Yan H., You Q., Yi X., Du Z., Xu W., Su Z. (2017). agriGO v2.0: A GO analysis toolkit for the agricultural community, 2017 update. Nucleic Acids Res..

[74] Kanehisa M., Furumichi M., Sato Y., Ishiguro-Watanabe M., Tanabe M. (2021). KEGG: Integrating viruses and cellular organisms. Nucleic Acids Res..

[75] Livak K.J., Schmittgen T.D. (2001). Analysis of relative gene expression data using real-time quantitative PCR and the 2(-Delta Delta C(T)) Method. Methods.

