# Supplementary material for: Transcriptomic Analysis Revealed Key Defense Genes and Signaling Pathways Mediated by the Arabidopsis thaliana Gene SAD2 in Response to Infection with Pseudomonas syringae pv. Tomato DC3000
Source: Int J Mol Sci. 2023 Feb 20;24(4):4229. doi: 10.3390/ijms24044229 (PMC9963955; doi:10.3390/ijms24044229)
Supplement: Supplementary file 1 [file ijms-24-04229-s001.zip › Figure S1.pdf]

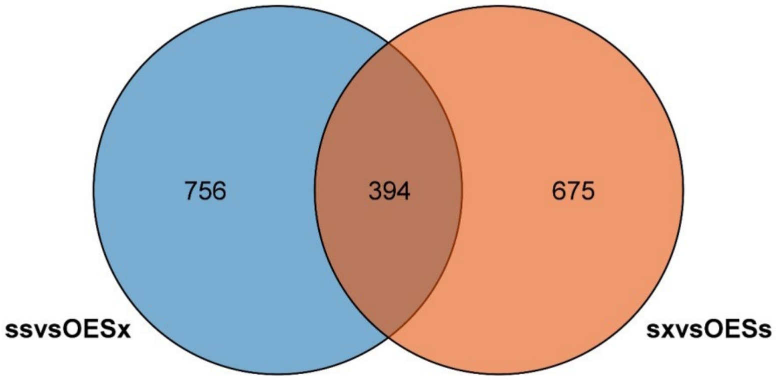

Figure S1 1825 differentially expressed genes (DEGs) presumed to be pathogen defense-related. ssvsOESx represents all the DEGs in Figure 3C, and sxvsOESs represents all the DEGs in Figure 3F.
